# Longitudinal course of sensory processing difficulties in pediatric obsessive–compulsive disorder: A preliminary naturalistic observational study

**DOI:** 10.1002/pcn5.70400

**Published:** 2026-08-26

**Authors:** Kenichi Yamane, Daisuke Katsuki, Yuki Iwaya, Yosuke Imamura, Eriko Nakatani, Tomohiro Nakao, Hiroshi Yamashita

**Affiliations:** ^1^ Department of Child and Adolescent Psychiatry Kyushu University Hospital Fukuoka Japan; ^2^ Department of Neuropsychiatry, Graduate School of Medicine Kyushu University Fukuoka Japan

**Keywords:** children and adolescents, clinical course, longitudinal study, obsessive–compulsive disorder, sensory processing difficulties

## Abstract

**Aim:**

Sensory processing difficulties (SPDs) are common in pediatric obsessive–compulsive disorder (OCD), but their longitudinal course remains undercharacterized. This retrospective naturalistic observational study examined 25 children and adolescents with OCD, including eight with comorbid autism spectrum disorder (ASD), who received specialist outpatient care in Japan. Analyses according to ASD status were exploratory.

**Methods:**

The study was designed to characterize longitudinal changes in caregiver‐rated SPDs rather than to evaluate treatment efficacy. Baseline and follow‐up assessments included the Short Sensory Profile–Japanese version (SSP‐J), Children's Yale–Brown Obsessive Compulsive Scale, and Children's Global Assessment Scale.

**Results:**

Caregiver‐rated SPDs decreased over time, with a significant main effect of time on SSP‐J scores (*F*(1, 23) = 5.66, *p* = 0.026, ηp^2^ = 0.197). SSP‐J scores were higher among participants with ASD across assessment points (*F*(1, 23) = 8.67, *p* = 0.007, ηp^2^ = 0.274), but the time × ASD interaction was not significant (*F*(1, 23) = 1.48, *p* = 0.236, ηp^2^ = 0.060). OCD severity decreased, and global functioning improved. Changes in SSP‐J scores were not significantly associated with changes in OCD severity or global functioning.

**Conclusion:**

In this sample, caregiver‐rated SPDs decreased over time. Participants with ASD had higher SSP‐J scores across assessment points, but the nonsignificant time × ASD interaction did not provide evidence of differential longitudinal change; ASD‐related findings remain exploratory. Changes in SPDs were not significantly associated with changes in OCD severity or global functioning, and the clinical value of direct assessment of sensory difficulties requires further study.

## INTRODUCTION

Obsessive–compulsive disorder (OCD) in childhood and adolescence is associated with substantial distress, avoidance, family accommodation, and functional impairment. Pediatric OCD is also clinically heterogeneous in its age at onset, comorbidity, presentation, and course.^1^ Sensory processing difficulties (SPDs) have been increasingly recognized as a clinically meaningful dimension of pediatric OCD. Cross‐sectional studies have shown that sensory over‐responsivity is common among treatment‐seeking youth with OCD and is associated with greater clinical severity and functional impairment.^2^, ^3^ Subsequent work has extended these observations across obsessive–compulsive and anxiety‐related conditions, supporting the view that SPDs may represent an important but under‐assessed clinical dimension in pediatric OCD.^3^, ^4^, ^5^, ^6^ However, most previous studies have been cross‐sectional, and longitudinal changes in caregiver‐rated SPDs in pediatric OCD remain undercharacterized. Some sensory difficulties may vary with distress, avoidance, anxiety, or broader clinical status, whereas others may reflect more enduring individual characteristics. Therefore, it remains unclear whether SPDs change over time and whether such changes parallel those in OCD severity or global functioning. Longitudinal assessment of these domains may help clarify the clinical course of sensory difficulties in pediatric OCD.

Autism spectrum disorder (ASD) is relevant to this question because OCD and ASD frequently co‐occur, and youth with both conditions may show distinct clinical characteristics.^7^, ^8^, ^9^, ^10^ Atypical sensory processing is also commonly reported in ASD and may be associated with a range of emotional and behavioral difficulties.^11^, ^12^ Short Sensory Profile measures have been widely used to characterize sensory features in children with ASD, although their psychometric interpretation in ASD samples warrants caution.^13^, ^14^ These observations provide a rationale for the exploratory examination of ASD status in longitudinal studies of SPDs in pediatric OCD.

Accordingly, this retrospective naturalistic observational study examined longitudinal changes in caregiver‐rated SPDs among children and adolescents with OCD receiving routine specialist outpatient care in Japan. We hypothesized that caregiver‐rated SPDs would improve over the follow‐up period. Associations between changes in SPDs, OCD severity, and global functioning were also examined. Given the small size of the ASD subgroup, analyses according to ASD status were exploratory.

## METHODS

### Study design and setting

This retrospective naturalistic observational study was based on routinely collected clinical data from a single‐center specialist child and adolescent psychiatric outpatient service at Kyushu University Hospital, Japan. This study characterized longitudinal changes in caregiver‐rated SPDs under routine care and was not designed to evaluate treatment efficacy.

Potentially eligible cases were identified from the departmental clinical database for the observation period from March 14, 2019, to December 25, 2025. Baseline was defined as the initial routine clinical assessment at treatment initiation, at which time the Children's Yale–Brown Obsessive Compulsive Scale (CY‐BOCS), Short Sensory Profile–Japanese version (SSP‐J), and Children's Global Assessment Scale (CGAS) were completed. Follow‐up was defined as the last available routine assessment within the observation period, during which all three measures were re‐administered. The follow‐up duration was calculated in months as the interval between baseline and follow‐up.

### Participants

We included consecutive children and adolescents aged 6–15 years at first presentation to the service who met the following criteria: (i) a primary clinical diagnosis of OCD according to the Diagnostic and Statistical Manual of Mental Disorders, Fifth Edition (DSM‐5), (ii) attendance at the outpatient service during the observation period, and (iii) complete CY‐BOCS, SSP‐J, and CGAS data at both baseline and follow‐up. No additional clinical exclusion criteria were applied in this study. The sample size was determined by the number of consecutive eligible patients with complete paired assessments available during the observation period rather than by an a priori sample size calculation.

Comorbid ASD and attention‐deficit/hyperactivity disorder (ADHD) were identified by board‐certified child and adolescent psychiatrists using a best‐estimate clinical approach, in accordance with DSM‐5 criteria. This approach was based on longitudinal clinical evaluation, developmental history, caregiver interviews, direct clinical assessment of the child or adolescent, and review of collateral information when available. Baseline Social Responsiveness Scale, Second Edition (SRS‐2;^15^, ^16^) scores were used descriptively as a dimensional index of autistic traits and were not used to determine ASD diagnosis. ADHD comorbidity was recorded for descriptive purposes.

### Routine clinical care

Clinical care was individualized according to each patient's needs. Treatment as usual could include exposure and response prevention‐based cognitive behavioral therapy, pharmacotherapy, or both. No standardized intervention protocol was implemented. Treatment selection, sequencing, frequency, duration, medication choice, and dose adjustment were determined by the treating clinicians rather than by the study protocol.

### Outcome measures

Standardized assessments were performed at baseline and follow‐up as part of routine clinical care. The primary clinical domain of interest was caregiver‐rated SPDs, assessed using the SSP‐J total score.^17^ SSP‐J raw total scores were reverse‐scored such that higher transformed scores indicated greater SPDs.

OCD severity was assessed using the CY‐BOCS total score, which ranges from 0 to 40, with higher scores indicating more severe obsessive–compulsive symptoms. The CY‐BOCS has established reliability and validity in pediatric OCD and has also shown utility in youth with ASD and clinically significant obsessive–compulsive symptoms.^18^, ^19^, ^20^ Remission was defined a priori as a CY‐BOCS total score ≤12^21^ and was used as a descriptive measure of OCD symptom status at follow‐up.

Global functioning was assessed using the CGAS score, which ranges from 1 to 100, with higher scores indicating better functioning.^22^ The CY‐BOCS and CGAS were rated by treating child and adolescent psychiatrists as part of the routine clinical assessment, whereas the SSP‐J was completed by the primary caregiver at both time points.

The caregiver‐rated Japanese version of the SRS‐2 was used to assess autistic traits, with higher scores indicating more pronounced autistic traits.

### Statistical analysis

Demographic and clinical characteristics of the total sample were summarized using means and standard deviations or counts and percentages, as appropriate, and are presented in Table 1. Exploratory between‐group comparisons of demographic and study characteristics and baseline clinical scores according to ASD status were performed using Welch's *t* tests for continuous variables and Fisher's exact tests for categorical variables, and the results are presented in Table S1.

**Table 1:** Demographic and clinical characteristics of the total sample (*N* = 25).

Characteristic	Total (*N* = 25)
Demographics	
Age (years)	11.64 ± 2.10
Male sex	13 (52.0%)
ADHD comorbidity	4 (16.0%)
Comorbid ASD	8 (32.0%)
Follow‐up duration (months)	16.28 ± 10.90
Baseline clinical scores	
CY‐BOCS (OCD severity)	24.12 ± 3.50
SSP‐J (sensory processing)	64.52 ± 16.10
SRS‐2 total (autistic traits)	59.72 ± 13.60
CGAS (global functioning)	46.60 ± 5.45

*Note*: Values are presented as n (%) or mean ± SD. SSP‐J total scores are reverse‐scored for interpretability; higher values indicate greater sensory processing difficulties.

Abbreviations: ADHD, attention‐deficit/hyperactivity disorder; ASD, autism spectrum disorder; CGAS, Children's Global Assessment Scale; CY‐BOCS, Children's Yale–Brown Obsessive Compulsive Scale; OCD, obsessive–compulsive disorder; SD, standard deviation; SRS‐2, Social Responsiveness Scale, Second Edition; SSP‐J, Short Sensory Profile–Japanese version.

Longitudinal changes were examined using mixed‐design repeated‐measures analyses of variance (ANOVAs) for the SSP‐J, CY‐BOCS, and CGAS scores. Time (baseline vs. follow‐up) was entered as the within‐subject factor, and ASD status (ASD, *n* = 8; non‐ASD, *n* = 17) was entered as the between‐subject factor. The SSP‐J model was the primary longitudinal analysis, whereas the CY‐BOCS and CGAS models were secondary analyses. In each model, the main effect of time was interpreted as an overall longitudinal change, the main effect of ASD status represented the mean difference between groups across assessment points, and the time × ASD interaction tested whether longitudinal change differed according to ASD status. Effect sizes for the omnibus ANOVA effects were reported as partial eta‐squared (ηp^2^). Analyses according to ASD status were exploratory because of the small and unequal subgroup sizes.

For transparency, exploratory model‐based simple‐effect comparisons and sensitivity analyses are reported in Table S2. These include within‐group changes, between‐group comparisons at follow‐up, Bonferroni‐adjusted *p* values, standardized effect‐size estimates, and a Welch's *t* test sensitivity analysis for the follow‐up SSP‐J comparison. Given the small and unequal subgroup sizes, all ASD‐stratified simple‐effect comparisons and sensitivity analyses were considered exploratory and hypothesis‐generating. Differential longitudinal changes according to ASD status were evaluated using the omnibus time × ASD interaction rather than subgroup‐specific significance tests. Associations among longitudinal change scores were examined using Pearson correlation coefficients. Change scores were calculated such that positive values indicated clinical improvement: baseline minus follow‐up scores for SSP‐J and CY‐BOCS, and follow‐up minus baseline scores for CGAS. We examined the associations between changes in SSP‐J and CY‐BOCS scores, between changes in SSP‐J and CGAS scores, between changes in CY‐BOCS and CGAS scores, and between changes in SSP‐J and follow‐up duration.

Assumptions relevant to exploratory between‐group comparisons were examined using Shapiro–Wilk tests for normality and Levene's tests for homogeneity of variance. When Levene's test indicated unequal variances, Welch's *t* tests were used for sensitivity analyses. No formal correction for multiplicity was applied across the three omnibus ANOVA models or the exploratory correlation analyses. All statistical tests were two‐tailed, with *α* = 0.05.

Complete CY‐BOCS, SSP‐J, and CGAS data at both assessment points were required for inclusion; therefore, there were no missing values in the variables used in the longitudinal analyses. Statistical analyses were performed using IBM SPSS Statistics for Windows, version 28.0 (IBM Corp.). Figures were generated using R software (R Foundation for Statistical Computing).

### Ethical considerations

The study was approved by the Ethics Committee of Kyushu University Hospital (Approval No. 25027) and conducted in accordance with the Declaration of Helsinki and its later amendments. Given the retrospective design using existing clinical records, the requirement for written informed consent was waived by the Ethics Committee. An opt‐out notice was provided to the participants and their guardians via the departmental website.

## RESULTS

### Participant flow and baseline characteristics

During the observation period, 28 children and adolescents with a primary DSM‐5 diagnosis of OCD who met the age criterion and underwent baseline standardized assessment were identified as potentially eligible. Three patients were excluded because complete paired CY‐BOCS, SSP‐J, and CGAS data at baseline and follow‐up were unavailable. The final analytic sample comprised 25 participants, including 8 (32.0%) with comorbid ASD and 17 (68.0%) without ASD. Four participants (16.0%) had comorbid ADHD.

Demographic and clinical characteristics of the total sample are summarized in Table 1. Baseline and follow‐up descriptive statistics according to ASD status are presented in Table S1. The ASD and non‐ASD groups did not differ significantly in terms of age, sex distribution, ADHD comorbidity, follow‐up duration, baseline CY‐BOCS scores, or baseline SSP‐J scores. Participants with ASD had higher baseline SRS‐2 total scores than those without ASD (69.88 ± 13.10 vs. 54.94 ± 11.25, *p* = 0.017), and lower baseline CGAS scores (42.63 ± 2.72 vs. 48.47 ± 5.46, *p* = 0.002).

### Primary longitudinal analysis: SPDs

**Figure 1:**
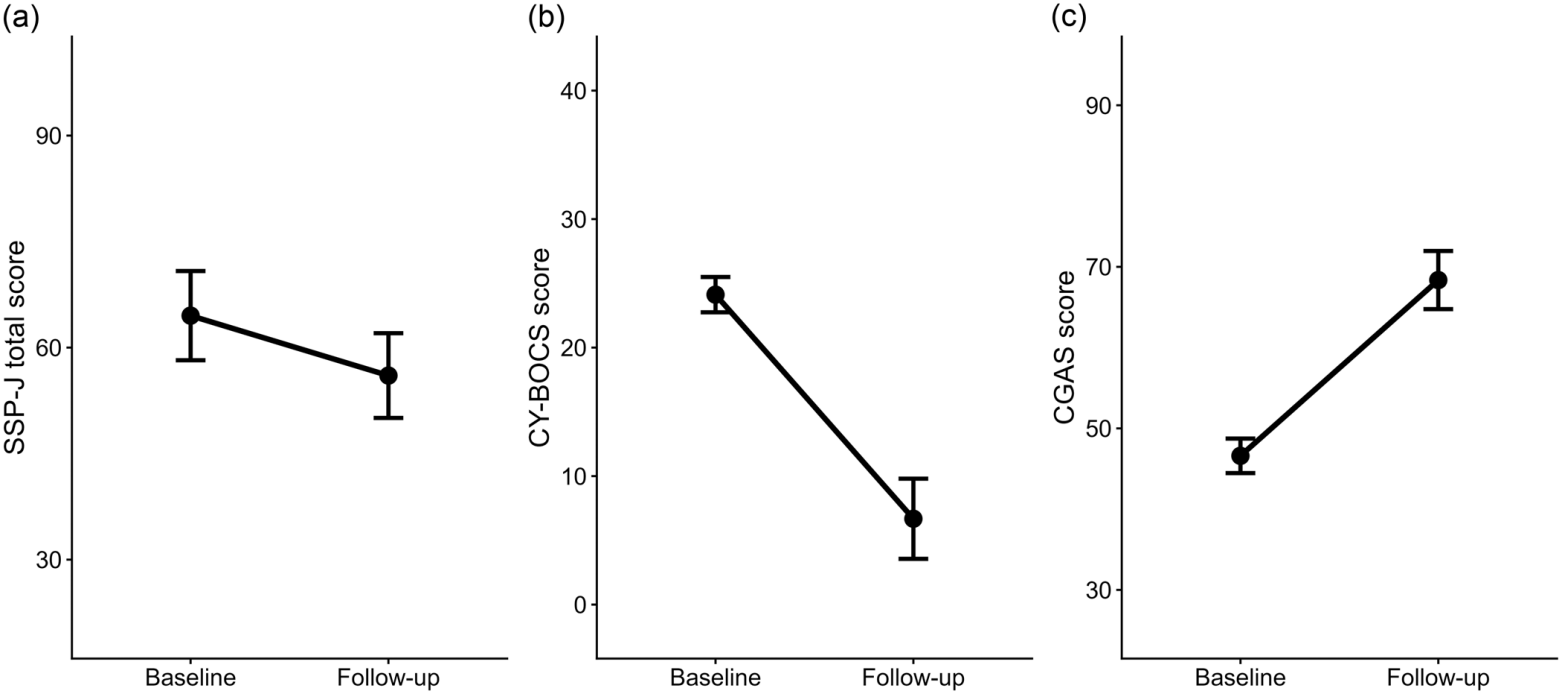
Longitudinal changes in sensory processing difficulties, obsessive–compulsive symptom severity, and global functioning in the full sample. (a) Mean reverse‐scored Short Sensory Profile–Japanese version (SSP‐J) total scores at baseline and follow‐up. Higher scores indicate greater sensory processing difficulties. (b) Mean Children's Yale–Brown Obsessive Compulsive Scale (CY‐BOCS) total scores at baseline and follow‐up. Higher scores indicate greater obsessive–compulsive symptom severity. (c) Mean Children's Global Assessment Scale (CGAS) scores at baseline and follow‐up. Higher scores indicate better global functioning. Error bars indicate 95% confidence intervals. Significant main effects of time were observed for the SSP‐J (*p* = 0.026), CY‐BOCS (*p* < 0.001), and CGAS (*p* < 0.001) in mixed‐design repeated‐measures analyses of variance.

SSP‐J total scores decreased from 64.52 ± 16.10 at baseline to 56.04 ± 15.29 at follow‐up, with a significant main effect of time (*F*(1, 23) = 5.66, *p* = 0.026, ηp^2^ = 0.197; Figure 1a). A significant main effect of ASD status was also observed (*F*(1, 23) = 8.67, *p* = 0.007, ηp^2^ = 0.274), indicating higher overall SSP‐J scores among participants with ASD across assessment points.

The time × ASD interaction was not statistically significant (*F*(1, 23) = 1.48, *p* = 0.236, ηp^2^ = 0.060) and did not provide evidence that longitudinal changes in SSP‐J scores differed according to ASD status. Baseline and follow‐up SSP‐J scores according to ASD status are presented in Table S1 and Figure S1a.

Exploratory model‐based simple‐effect comparisons and sensitivity analyses are reported in Table S2. Because the time × ASD interaction was not statistically significant, these supplementary analyses were not interpreted as evidence of differential longitudinal changes according to ASD status.

### Secondary longitudinal analysis: OCD severity

Mean CY‐BOCS total scores decreased from 24.12 ± 3.50 at baseline to 6.68 ± 7.96 at follow‐up, with a significant main effect of time (*F*(1, 23) = 116.44, *p* < 0.001, ηp^2^ = 0.835; Figure 1b). Neither the main effect of ASD status (*F*(1, 23) = 0.25, *p* = 0.624, ηp^2^ = 0.011) nor the time × ASD interaction (*F*(1, 23) = 0.01, *p* = 0.932, ηp^2^ < 0.001) was statistically significant. The nonsignificant interaction did not provide evidence that longitudinal changes in CY‐BOCS scores differed according to ASD status.

Baseline and follow‐up CY‐BOCS scores according to ASD status are presented in Table S1 and Figure S1b. At follow‐up, 20 participants (80.0%) met the predefined remission criterion of a CY‐BOCS total score ≤12.

### Secondary longitudinal analysis: Global functioning

Mean CGAS scores increased from 46.60 ± 5.45 at baseline to 68.36 ± 9.18 at follow‐up, with a significant main effect of time (*F*(1, 23) = 170.41, *p* < 0.001, ηp^2^ = 0.881; Figure 1c). A significant main effect of ASD status was observed (*F*(1, 23) = 10.95, *p* = 0.003, ηp^2^ = 0.323), indicating lower CGAS scores among participants with ASD across assessment points.

The time × ASD interaction was not statistically significant (*F*(1, 23) = 1.44, *p* = 0.242, ηp^2^ = 0.059) and did not provide evidence that longitudinal changes in CGAS scores differed according to ASD status. Baseline and follow‐up CGAS scores according to ASD status are presented in Table S1 and Figure S1c.

### Associations among longitudinal changes

Improvement in global functioning was moderately associated with a reduction in OCD severity (*r* = 0.486, *p* = 0.014). Changes in SSP‐J total scores were not significantly correlated with changes in CY‐BOCS scores (*r* = −0.120, *p* = 0.568) or with changes in CGAS scores (*r* = 0.102, *p* = 0.627). Changes in SSP‐J scores were also not significantly associated with follow‐up duration (*r* = 0.282, *p* = 0.171).

## DISCUSSION

In this preliminary retrospective naturalistic study, caregiver‐rated SPDs decreased from baseline to follow‐up in children and adolescents with obsessive–compulsive disorder (OCD). Changes in the Short Sensory Profile–Japanese version (SSP‐J) scores were not significantly associated with changes in OCD severity or global functioning, whereas a reduction in OCD severity was associated with improvement in global functioning. Participants with comorbid ASD had higher SSP‐J scores on average across assessment points; however, the time × ASD interaction was not statistically significant. Thus, the present data do not show that sensory difficulties follow different longitudinal courses according to ASD status. Because only eight participants had ASD, all ASD‐related estimates should be regarded as exploratory and hypothesis‐generating.

The longitudinal decrease in SSP‐J scores extends previous cross‐sectional findings that sensory difficulties are common in pediatric OCD and related conditions.^2^, ^3^, ^4^, ^6^ The present observation indicates that caregiver‐rated sensory difficulties may change during clinical follow‐up rather than remain entirely stable. Possible contributors include changes in distress, avoidance, daily routines, environmental demands, developmental adaptation, or caregiver appraisal. Because the participants received heterogeneous routine care and no comparison group was included, the observed decrease could not be attributed to a specific intervention or mechanism. Moreover, the absence of significant correlations between changes in SSP‐J scores and changes in CY‐BOCS or CGAS scores does not establish that these domains are independent; modest associations may not have been detectable in this small sample.

The higher average SSP‐J scores among participants with ASD are consistent with the broader literature describing sensory processing differences in ASD.^11^, ^12^ Importantly, however, a between‐group main effect describes the average difference across assessment points and does not demonstrate a difference in the magnitude or direction of longitudinal change. The current findings, therefore, do not support the conclusion that SPDs improved less, persisted, or represented a residual sensory burden in the ASD group. Estimates from the ASD subgroup were imprecise and may have been strongly influenced by individual observations. Larger samples with adequate power to test the time × ASD interaction are required before conclusions regarding ASD‐related longitudinal differences can be drawn.

OCD severity decreased, and global functioning improved during follow‐up, and these changes were moderately associated. The finding that 80.0% of participants met the predefined remission criterion provides a descriptive context for the clinical status of this cohort at follow‐up. However, the complete‐case analysis, variable follow‐up duration, and inclusion of patients who remained engaged in specialist care may have influenced the magnitude of the observed improvement. Therefore, these outcomes should be interpreted descriptively as features of the observed clinical course.

In this sample, changes in caregiver‐rated SPDs did not significantly covary with changes in OCD symptoms or global functioning; whether repeated sensory assessments provide additional information during follow‐up is unclear. This result does not establish a separate causal process or the effectiveness of a sensory‐specific intervention. Nevertheless, when sensory difficulties contribute to distress or functional impairment, individualized assessments may inform caregiver psychoeducation, environmental adjustments, school coordination, and adaptation of psychological interventions to the child's needs.

This study has several strengths, including paired baseline and follow‐up assessments, the use of standardized measures across sensory, symptom, and functional domains, and observation in a routine specialist setting. This study also has several important limitations. The total sample size was small, and the ASD subgroup included only eight participants, limiting precision and statistical power. The retrospective single‐center design restricts generalizability and may have introduced selection bias through the complete‐case requirement. The follow‐up intervals and clinical care were heterogeneous, and the treatment type and intensity could not be modeled. ASD and ADHD diagnoses were based on best‐estimate clinical assessments rather than standardized research instruments. CY‐BOCS and CGAS ratings were completed by treating clinicians without blinding, whereas SPDs were assessed only by caregiver reports. The use of two assessment points precluded the evaluation of nonlinear change or temporal ordering, and multiplicity was not formally controlled across the omnibus and correlation analyses. Finally, the observation window spanned the COVID‐19 pandemic, and unmeasured calendar‐related changes in clinical service delivery, school environment, and family functioning may have influenced the observed changes.

Future multicenter prospective studies should include larger samples, standardized assessments of ASD, repeated assessments at prespecified intervals, and multi‐informant or performance‐based measures of sensory functioning. Detailed measurement of treatment exposure and environmental changes would help clarify the factors associated with sensory changes. Most importantly, adequately powered analyses of the time × ASD interaction are needed to determine whether the longitudinal patterns of SPDs differ according to ASD status.

## CONCLUSION

In this preliminary naturalistic study of pediatric OCD, caregiver‐rated SPDs decreased during follow‐up; however, changes in SPDs were not significantly associated with changes in OCD severity or global functioning. Participants with ASD had higher SSP‐J scores on average across assessment points, but the nonsignificant time × ASD interaction did not provide evidence that longitudinal changes differed according to ASD status. These findings support further longitudinal studies of sensory difficulties as a clinically relevant dimension of pediatric OCD, while ASD‐related observations should remain exploratory until confirmed in larger prospective samples.

## AUTHOR CONTRIBUTIONS

Kenichi Yamane and Daisuke Katsuki conceived and designed the study, collected the clinical data, and drafted the manuscript. Yuki Iwaya and Yosuke Imamura contributed to data curation. Daisuke Katsuki performed the statistical analyses. Eriko Nakatani, Tomohiro Nakao, and Hiroshi Yamashita supervised the study and provided methodological and clinical input. All authors critically reviewed and approved the final manuscript.

## CONFLICT OF INTEREST STATEMENT

The authors declare no conflicts of interest.

## ETHICS APPROVAL STATEMENT

All procedures performed in this study involving human participants were in accordance with the ethical standards of the institutional research committee (Ethics Committee of Kyushu University Hospital; Approval No. 25027) and with the Declaration of Helsinki and its later amendments.

## PATIENT CONSENT STATEMENT

The requirement for written informed consent was waived by the Ethics Committee of Kyushu University Hospital owing to the retrospective nature of the study utilizing medical records. Instead, an opt‐out notice was posted on the department's website, giving participants and/or their guardians the opportunity to decline the use of their data.

## CLINICAL TRIAL REGISTRATION

N/A.

## DECLARATION OF GENERATIVE AI AND AI‐ASSISTED TECHNOLOGIES IN THE MANUSCRIPT PREPARATION PROCESS

During the preparation of this manuscript, the authors used AI‐assisted tools only to support language editing and improve readability. These tools were not used to generate data, perform analyses, or formulate scientific conclusions. The authors have reviewed and approved all revisions and take full responsibility for the final content of the manuscript.

## Supporting information


**Supplementary Fig. S1** Longitudinal changes in sensory processing difficulties, OCD severity, and global functioning by ASD status. (A) Mean Short Sensory Profile–Japanese version (SSP‐J) total scores at baseline and follow‐up by comorbid autism spectrum disorder (ASD) status. SSP‐J scores are reverse‐scored, such that higher scores indicate greater sensory processing difficulties. (B) Mean Children's Yale–Brown Obsessive Compulsive Scale (CY‐BOCS) total scores at baseline and follow‐up by ASD status. Higher CY‐BOCS scores indicate more severe obsessive–compulsive symptoms. (C) Mean Children's Global Assessment Scale (CGAS) scores at baseline and follow‐up by ASD status. Higher CGAS scores indicate better global functioning. Error bars indicate 95% confidence intervals for group means. Significant main effects of time were observed for all three outcomes. No significant time × ASD status interaction was observed for any outcome. Significant main effects of ASD status were observed for SSP‐J and CGAS, but not for CY‐BOCS.

Supporting File 1.

## Data Availability

The datasets generated and/or analyzed during the current study are not publicly available because of ethical and privacy restrictions involving sensitive clinical information from minors. De‐identified data may be made available from the corresponding author upon reasonable request, subject to approval by the Ethics Committee of Kyushu University Hospital and applicable institutional regulations.
