# Supplementary material for: Longitudinal course of sensory processing difficulties in pediatric obsessive–compulsive disorder: A preliminary naturalistic observational study
Source: PCN Rep. 2026 Aug 26;5(3):e70400. doi: 10.1002/pcn5.70400 (PMC13508018; doi:10.1002/pcn5.70400)
Supplement: Supplementary file 1 [file PCN5-5-e70400-s001.docx]

**Supplementary Tables**

**Supplementary Table S1. Demographic and clinical characteristics and baseline and follow-up scores by ASD status (n = 25)**

| **Characteristic** | **Total (N = 25)** | **ASD group (n = 8)** | **Non-ASD group (n = 17)** | ***p* value** |
| --- | --- | --- | --- | --- |
| **Demographic and study characteristics** | | | | |
| Age (years) | 11.64 ± 2.10 | 11.63 ± 2.62 | 11.65 ± 1.90 | 0.983 |
| Male sex | 13 (52.0%) | 5 (62.5%) | 8 (47.1%) | 0.673 |
| ADHD comorbidity | 4 (16.0%) | 2 (25.0%) | 2 (11.8%) | 0.570 |
| Follow-up duration (months) | 16.28 ± 10.90 | 15.00 ± 7.07 | 16.88 ± 12.45 | 0.636 |
| SRS-2 total, baseline (autistic traits) | 59.72 ± 13.60 | 69.88 ± 13.10 | 54.94 ± 11.25 | 0.017 |
| **Clinical scores at baseline and follow-up** | | | | |
| *SSP-J (sensory processing difficulties)* | | | | |
| Baseline | 64.52 ± 16.10 | 72.50 ± 18.71 | 60.76 ± 13.74 | 0.142 |
| Follow-up | 56.04 ± 15.29 | 69.00 ± 18.34 | 49.94 ± 8.96 | — |
| *CY-BOCS (OCD severity)* | | | | |
| Baseline | 24.12 ± 3.50 | 24.75 ± 3.20 | 23.82 ± 3.70 | 0.530 |
| Follow-up | 6.68 ± 7.96 | 7.50 ± 7.80 | 6.29 ± 8.24 | — |
| *CGAS (global functioning)* | | | | |
| Baseline | 46.60 ± 5.45 | 42.63 ± 2.72 | 48.47 ± 5.46 | 0.002 |
| Follow-up | 68.36 ± 9.18 | 61.75 ± 4.65 | 71.47 ± 9.21 | — |

Note: Values are presented as n (%) or mean ± SD. SSP-J total scores are reverse-scored for interpretability; higher values indicate greater sensory processing difficulties. The *p* values shown are from Welch’s t tests for continuous variables and Fisher’s exact tests for categorical variables. Follow-up values are presented descriptively only, and no between-group significance tests are reported for them (—); differential longitudinal change according to ASD status was evaluated using the omnibus time × ASD interaction in the mixed-design repeated-measures analyses of variance reported in the main text, which was not statistically significant for any measure. Given the small and unequal subgroup sizes, all ASD-stratified estimates are exploratory.

Abbreviations: ADHD, attention-deficit/hyperactivity disorder; ASD, autism spectrum disorder; CGAS, Children’s Global Assessment Scale; CY-BOCS, Children’s Yale–Brown Obsessive Compulsive Scale; OCD, obsessive–compulsive disorder; SD, standard deviation; SRS-2, Social Responsiveness Scale, Second Edition; SSP-J, Short Sensory Profile–Japanese version.

**Supplementary Table S2.** Exploratory model-based simple-effect comparisons and sensitivity analyses for SSP-J scores by ASD status

| **Comparison** | **Estimate** |
| --- | --- |
| Follow-up: ASD vs. non-ASD group, mean difference (95% CI) | 19.06 (7.91 to 30.21) |
| Follow-up: ASD vs. non-ASD group, omnibus-model simple effect | F(1, 23) = 12.50, Bonferroni-adjusted *p* = .002 |
| Follow-up: ASD vs. non-ASD group, sensitivity analysis | Welch’s t(8.61) = 2.79, *p* = .022 |
| Follow-up: ASD vs. non-ASD group, standardized effect size | Cohen’s d = 1.51; Hedges’ g = 1.46 |
| Within-group change, baseline to follow-up (ASD group, n = 8) | Bonferroni-adjusted *p* = .488, ηp^2^ = .021 |
| Within-group change, baseline to follow-up (non-ASD group, n = 17) | Bonferroni-adjusted *p* = .004, ηp^2^ = .305 |

Note: Analyses are restricted to Short Sensory Profile–Japanese version (SSP-J) total scores, the focus of the primary longitudinal analysis. Because the omnibus time × ASD interaction for SSP-J was not statistically significant, these model-based simple-effect and sensitivity analyses are exploratory and hypothesis-generating and should not be interpreted as evidence of differential longitudinal changes according to ASD status. Given the small ASD subgroup (n = 8), effect-size and p-value estimates are imprecise and may be sensitive to individual observations. The *p* values for within-group and between-group simple-effect comparisons were Bonferroni-adjusted for multiple comparisons within each omnibus model. Abbreviations: ASD, autism spectrum disorder; CI, confidence interval; SSP-J, Short Sensory Profile–Japanese version.
